# Chest X-ray Foreign Objects Detection Using Artificial Intelligence

**DOI:** 10.3390/jcm12185841

**Published:** 2023-09-08

**Authors:** Jakub Kufel, Katarzyna Bargieł-Łączek, Maciej Koźlik, Łukasz Czogalik, Piotr Dudek, Mikołaj Magiera, Wiktoria Bartnikowska, Anna Lis, Iga Paszkiewicz, Szymon Kocot, Maciej Cebula, Katarzyna Gruszczyńska, Zbigniew Nawrat

**Affiliations:** 1 Department of Biophysics, Faculty of Medical Sciences in Zabrze, Medical University of Silesia, Jordana 19, 41-808 Zabrze, Poland; znawrat@sum.edu.pl; 2 Paediatric Radiology Students’ Scientific Association at the Division of Diagnostic Imaging, 40-752 Katowice, Poland; katarzyna.bargiel@op.pl (K.B.-Ł.); wiktoriabart96@gmail.com (W.B.); 3 Department of Radiology and Nuclear Medicine, Faculty of Medical Sciences in Katowice, Medical University of Silesia, 40-752 Katowice, Poland; kgruszczynska@sum.edu.pl; 4 Division of Cardiology and Structural Heart Disease, Medical University of Silesia, 40-635 Katowice, Poland; kozlik.maciej@gmail.com; 5 Professor Zbigniew Religa Student Scientific Association at the Department of Biophysic, Faculty of Medical Sciences in Zabrze, Medical University of Silesia, Jordana 19, 41-808 Zabrze, Poland; lukczog@gmail.com (Ł.C.); piotrekd233@gmail.com (P.D.); mikaczu7422@gmail.com (M.M.); igapaszkiewicz.ip@gmail.com (I.P.); 6 Cardiology Students’ Scientific Association at the III Department of Cardiology, Faculty of Medical Sciences in Katowice, Medical University of Silesia, 40-635 Katowice, Poland; lis.anna9898@gmail.com; 7 Bright Coders’ Factory, Technologiczna 2, 45-839 Opole, Poland; szymko1995@gmail.com; 8 Individual Specialist Medical Practice, 40-754 Katowice, Poland; maciejmichalcebula@gmail.com; 9 Foundation of Cardiac Surgery Development, 41-800 Zabrze, Poland

**Keywords:** artificial intelligence, artifacts, chest X-ray, convolutional neural network, foreign body

## Abstract

Diagnostic imaging has become an integral part of the healthcare system. In recent years, scientists around the world have been working on artificial intelligence-based tools that help in achieving better and faster diagnoses. Their accuracy is crucial for successful treatment, especially for imaging diagnostics. This study used a deep convolutional neural network to detect four categories of objects on digital chest X-ray images. The data were obtained from the publicly available National Institutes of Health (NIH) Chest X-ray (CXR) Dataset. In total, 112,120 CXRs from 30,805 patients were manually checked for foreign objects: vascular port, shoulder endoprosthesis, necklace, and implantable cardioverter-defibrillator (ICD). Then, they were annotated with the use of a computer program, and the necessary image preprocessing was performed, such as resizing, normalization, and cropping. The object detection model was trained using the You Only Look Once v8 architecture and the Ultralytics framework. The results showed not only that the obtained average precision of foreign object detection on the CXR was 0.815 but also that the model can be useful in detecting foreign objects on the CXR images. Models of this type may be used as a tool for specialists, in particular, with the growing popularity of radiology comes an increasing workload. We are optimistic that it could accelerate and facilitate the work to provide a faster diagnosis.

## 1. Introduction

Diagnostic imaging has become an essential part of the healthcare system, and its accuracy is crucial for undertaking proper treatment. However, as the number of radiological examinations increases, the workload of specialists increases. Chest X-rays represent 40% of the 3.6 billion imaging procedures performed worldwide every year [1]. In the United States (US), more than 70 million CXR examinations are performed annually [2]. Artificial intelligence (AI) has received considerable attention over the past decade. Deep learning (DL) is a subcategory of AI and is based on deep neural networks (DNNs). The element that differentiates DNNs from artificial neural networks (ANNs) is the use of multiple hidden layers in a network’s architecture. This enables the network to understand and mimic more complex behaviors and processes [3]. As an example of the use of DNNs, Ghaderzadeh et al. created a computer-aided detection (CAD) system for COVID-19 on CT scans. The proposed model achieved an accuracy of 99.6% on the test dataset [4]. In other research carried out in another field, Ghaderzadeh et al. developed a CNN-based model able to detect ALL from hematogone cases and assist hematologists and laboratory personnel in diagnosing ALL subtypes. Peripheral blood smear was used as the imaging material in this study. The model based on DenseNet201 accomplished sensitivity, specificity, and accuracy on the level over 99% on the test dataset [5]. Other hematology research conducted by Hosseini et al. established a model and mobile application for screening B-ALL cases from non-B-ALL cases. Accuracy, sensitivity, and specificity of the DNN-based model reached 100% in both test dataset and in laboratory settings [6]. Research by Garavand et al. from the other side of the diagnostic spectrum aimed to review and evaluate machine learning methods in the diagnosis of coronary artery disease. Laboratory and demographic data classification has been used by AI systems such as traditional ML, DL/NN, and ensemble. Of the 54 studies included in the review, an impressive 24 were studies based on the use of DNNs [7].

Motivated by the success of artificial intelligence, especially deep learning in medical image analysis, researchers around the world are working on AI-based tools to help radiologists in the diagnostic process. A big part of this is the error-free nature of artificial intelligence. One of the reasons that make radiological image evaluation difficult is artifacts. These are image fragments that are not anatomical structures caused by hardware defects, including software error, operator error, or foreign bodies (e.g., ICDs [8], ECG leads, jewelry [9], endotracheal tubes, cardiac clips [10], coins [11], or silicone breast implants [12]) inside or outside the patient’s body, as well as patient movement during the examination (motion artifacts). A separate radiographic imaging method should be chosen for each foreign body, depending on the material and its depth and location in the body. Metal and glass bodies are easily detectable, unlike wooden ones. In studies using X-rays, ultrasound or CT-scan is recommended if the location is inaccurate or the presence and characteristics of the foreign body are inconclusive [13]. Baram et al. proved that more than 50% of imaging studies with foreign bodies in the trachea and 25% of foreign bodies in the bronchi were not detected on X-rays [14]. Another example is the misinterpretation of ECG electrodes, or a ruptured silicone implant [15], considered to be pulmonary nodules on chest X-rays in AP projection, which was confirmed by a follow-up X-ray in lateral projection [16,17,18]. Artifacts can also hinder diagnosis by obscuring organs. An example is endotracheal tubes, which, when placed incorrectly, can cause health complications [19], as well as hinder the diagnostic process. This is especially useful in intensive care units where the speed of accurately identifying its placement is often critical for the patient [20]. Neural network-based systems are also now being used to recognize and classify implanted devices such as pacemakers/loop recorders/defibrillators (Implantable cardioverter-defibrillators, ICDs) visible on chest X-rays. This is particularly important in clinical emergencies that require medical personnel to act immediately. The presence of ICD devices on the images can be considered an artifact in the form of an object but can also provide a lot of relevant clinical information [21]. This information can be provided by the patient themself and is also contained in the medical records of the ICD device. If it is not possible to obtain them, specialized personnel can distinguish the ICD model on an X-ray, but the method is not perfect and often poses difficulties [22]. For this reason, artificial intelligence algorithms are eagerly becoming involved in the process, shortening it considerably, with accuracy in identifying the manufacturer, and models reaching 99.6% [8]. Applications for mobile devices are also being developed to further facilitate the process for clinicians [21]. Examples of devices a radiologist may encounter include shoulder joint endoprostheses [23,24], bowel motility markers, gastric banding, abdominal tubes [25], neurostimulator endoprostheses, hemostatic cellulose gauze, vascular bypass [26], intra-aortic balloon pumps [26], ventriculoperitoneal valves [26,27], tracheobronchial stents [26], coronary artery stents [26,27], ventricular assist devices [26,27,28], endoscopic capsules, endobronchial devices, devices used in prostate brachytherapy, or hernia meshes [25,26]. In the following study, AI was used to determine selected artifacts on X-ray images. The focus was on detection by a neural network of objects such as an ICD, a necklace, a shoulder joint endoprosthesis, and a vascular port. It has been proven that AI using a convolutional neural network (CNN) can recognize pathologies on X-ray images with comparable efficiency to radiologists [29].

## 2. Materials and Methods

### 2.1. Radiological Part

**Figure 1:**
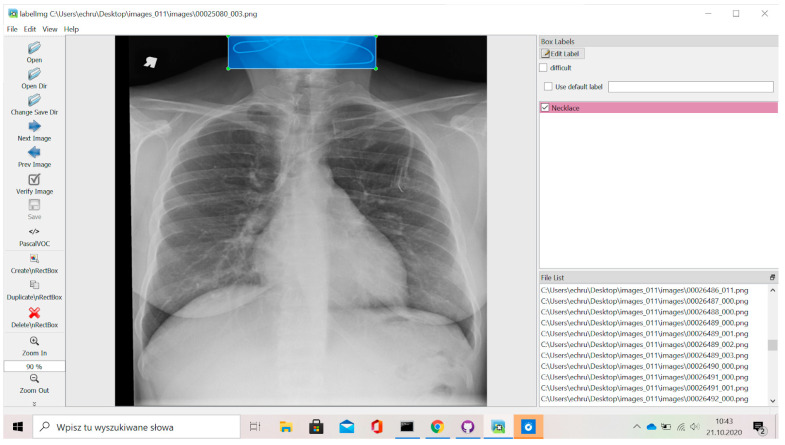
Presentation of the labeling process in the LabelImg program. The image shows a selected necklace (structure in the blue area) with the appropriate color of the label.

For the present study, the NIH Chest X-ray Dataset was used. The dataset contains 112,120 X-ray images of the scans of 30,805 unique individuals [30]. To create these labels, the authors used natural language processing to extract disease classifications via text from relevant radiology reports. The labels are over 90% well-picked and are expected to be suitable for weakly supervised learning. Five researchers using the LabelImg program (Figure 1) marked a total of 9937 foreign objects on chest X-rays, such as vascular port (7648), necklace (1613), ICD (498), and shoulder joint endoprosthesis (178). Markings were made in the form of boxes that covered the area of interest—the foreign objects. The dataset created was validated twice by a specialist radiologist. After the validation process, corrections were also applied twice in order to obtain better quality labels. The corrections were made by people other than those who originally performed the labeling.

### 2.2. Technical Part

The location of the box on the chest X-ray showing the foreign object was then encoded as coordinates, e.g., [880.0, 435.0, 1004.0, 532.0] (Figure 1). The object detection model was trained using the YOLOv8 architecture and the Ultralytics framework [26]. The dataset used for training and evaluating the models consisted of 8703 images of 1024 × 1024 pixels with 1 or more foreign objects of the following classes: Endo (shoulder joint endoprosthesis), ICD (implantable cardioverter-defibrillator), Necklace (neck jewelry), and Port (vascular port), which have been divided into training and validation subsets using a 90:10 split. The YOLO v8 architecture is a real-time object detection model. This is the latest version of the popular algorithm for fast detection of objects in images. YOLO v8 was released in 2021 and includes several improvements over previous versions such as better accuracy, faster performance, and increased flexibility. It uses a single convolutional network to predict object boundaries and class probabilities, directly from complete images.

The training was conducted on a single Nvidia A100 GPU using the Google Colab architecture. The models were trained using the Stochastic Gradient Descent (SGD) optimizer with a training rate of 0.01 and a batch size of 64. The training process was stopped after 100 epochs, when the validation loss plateaued. Data augmentation was used to increase the diversity of the training dataset. This included random horizontal flips, random scaling, and random framing. During training, model performance was assessed using the Mean Average Precision (mAP) metric. The final model was selected based on the mAP score on the validation set. The models were implemented in Pytorch and trained using the Ultralytics YOLOv8 library and a nano variant of the model using transfer learning. The model consisted of 225 layers and 3.01 M parameters.

## 3. Results

**Figure 2:**
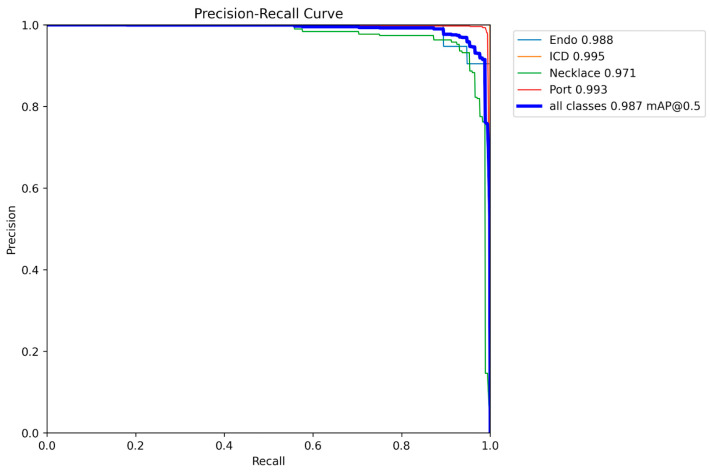
The graph shows the Precision–Recall Curve for each of the foreign objects classes (endo, ICD, necklace, and port) as well as for all the classes. The mean average precision at intersection over union threshold equal to 0.5 was calculated.

For the evaluation of our model, the average precision (AP) for Intersection over Union (IoU) from 0.5 to 0.95 was used. The step size was set as 0.05. The trained object-detection model achieved an impressive AP of 0.815 on the validation set, indicating high object detection accuracy. The model was able to accurately detect and classify a wide range of object classes, including necklaces, vascular ports, ICDs, and shoulder joint replacements. The average AP for the IoU threshold of 50–95% (mAP 50–95) was: Endo—0.859, ICD—0.823, Necklace—0.79, and Port—0.789. The value for all foreign objects was 0.815. The Area under the Precision–Recall curve at 50% IoU threshold: Endo—0.988, ICD—0.995, Necklace—0.971, and Port—0.993. The value for all foreign objects was equal to 0.987 (Figure 2).

**Figure 3:**
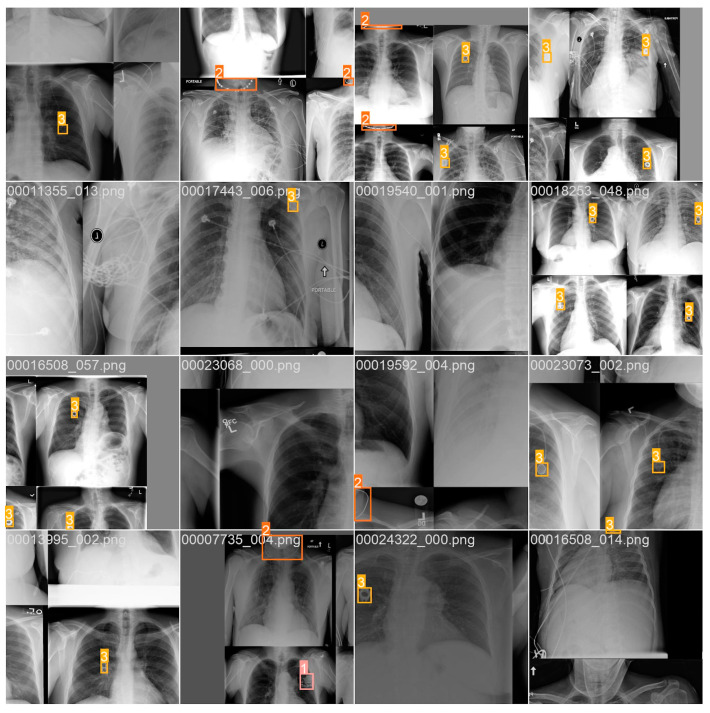
Sample-labeled images used to test the model. Rectangle-shaped and square-shaped areas in yellow, orange, and pink colors are areas with marked foreign objects (bounding boxes).

A qualitative analysis of the model’s predictions showed that the model was able to accurately detect and classify objects even on tests that were particularly difficult to analyze, such as overexposed, shaded, not covering the full examined area or presented incorrectly. Some images have been specially modified so that their quality differs significantly from standard X-rays (Figure 3).

**Figure 4:**
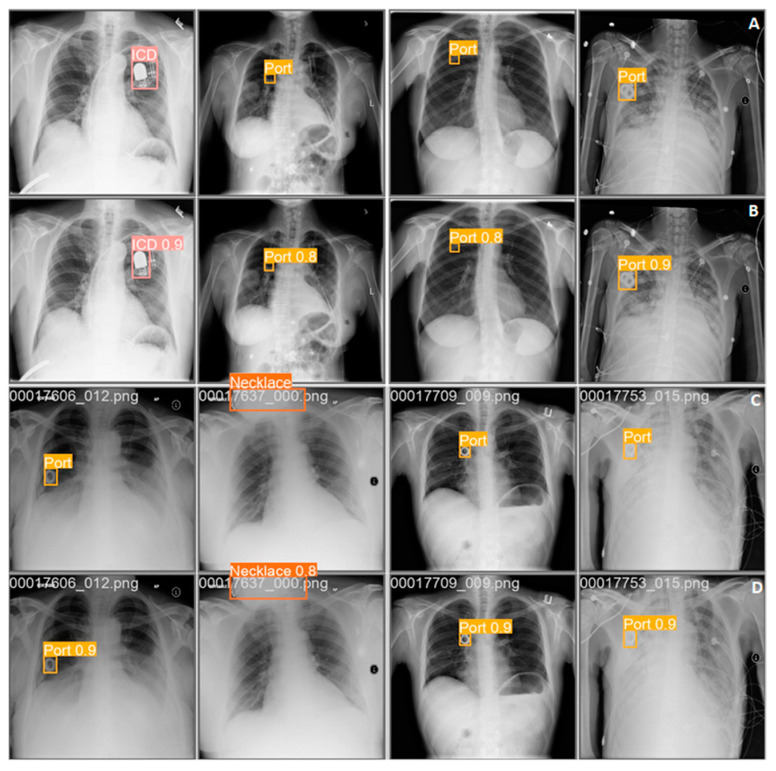
The image shows a comparison of the placement of bounding boxes (rectangular-shaped and square-shaped areas in pink, yellow, and orange colours) labeled by the model and the annotator evaluating the X-ray images. Rows A and C represent images evaluated by the annotator, whereas rows B and D were labeled by the AI.

In addition, the performance of the real-time model was evaluated by measuring the prediction time on the Nvidia A100. The model was able to process 250 images per second. The predictions obtained by the network reflected the actual locations of foreign objects. The comparison of the results of the network and the determinations made by the researchers is shown in (Figure 4).

For verification, images with the neural network’s boxes overlaid on chest X-rays were generated to visually assess the correctness of the network (Figure 4). A total of 100 randomly selected chest X-ray images with artifacts were generated and manually checked for the correctness of the type of foreign body and the correctness of the box marking by the AI. All the 100 randomly selected chest X-ray images checked had the type and boxed area correctly applied.

## 4. Related Works

The use of a variety of algorithms is gaining increasing application in various fields of medicine, especially radiology. AI-based solutions can be applied, for example, in detecting pathologies or different objects on CXRs. The study from Kara et al. showed high accuracy for both the identification and localization of endotracheal tube tips and carinae on chest X-ray images [20]. Moreover, most common diseases like pneumonia can be detected by an algorithm—Rajpurkar et al. developed an algorithm called CheXNet that can detect pneumonia from chest X-rays at a level exceeding practicing radiologists [29]. There is also a growing interest in creating mobile phone applications that help physicians in everyday work, like in Weinreich et al.’s study on an application for identification of cardiac implanted electrical devices (CIEDs) (e.g., pacemakers and defibrillators) in urgent or emergent settings [21]. Furthermore, as CIEDs become more and more common, it is useful to develop and evaluate a deep learning-based algorithm that performs the detection and characterization of parameters, including MRI safety, of CIEDs on chest X-ray images, like Kim et al. performed [31]. Comparisons of different machine learning algorithms are also available—for example, Chudow et al. compared algorithms for identification of CIEDs using chest radiography: The PacemakerID algorithm, available as a mobile phone application (PIDa) and a web platform (PIDw), and The Pacemaker Identification with Neural Networks (PPMnn), available via web platform [22]. However, AI does not only support diagnostic processes; it can also help when planning and carrying out therapy. Sultan et al. proposed three different deep learning-based frameworks to identify different types of shoulder implants in X-ray scans, mainly an ensemble network called the Inception Mobile Fully Connected Convolutional Network (IMFC-Net) [23]. Only selected examples are listed here, but they illustrate how broadly AI-based algorithms can be used.

## 5. Discussion

Over the last decades, many new devices and elements of the instrumentation were developed and have become a permanent part of the diagnostic images described by radiologists. These tools are often implanted in the patient. An example are the ones placed on the patient’s body surface in order to perform specific tests—clips, cables, or ECG electrodes [32] or others inside the body, such as endovascular occlusion devices, thrombolysis catheters [27]. The widespread use of X-rays as a diagnostic tool is associated with the equally frequent occurrence of the above-mentioned devices as elements of radiographs.

The model proposed in this study achieved an impressive average precision and high object detection accuracy and was able to classify foreign bodies in four classes. This shows that it can contribute to a decrease in the number of missed foreign bodies on X-ray images. Thus, these detected foreign objects would not be mistaken for pathologies and other artifacts. In the training process, the photos had been specially transformed to imitate images of lower quality and difficult to analyze, such as can be found in everyday clinical practice (for instance bed-side chest X-ray). This clearly proves its usefulness in a clinical environment. Moreover, it was amazingly fast (250 images/s), which would also contribute to clinical practice.

The rapid and efficient detection of foreign bodies is also highly useful for various reasons. The strong magnetic field generated by magnetic resonance imaging (MRI), among other things, poses a danger to the health and life of patients with implanted devices made of metal, such as ICDs and endoprostheses. Performing such an examination on a patient with an implanted metal device can lead to, among other things, abnormal heart rhythms, inhibited pacing, or heating up or even damage to the entire device. Most implanted devices used in cardiology are contraindications for diagnostic tests using magnetic fields. An exception is the modern SureScan devices [33], which are approved for MRI. Hence, the conclusion is that detection of metallic components increases safety when classifying a patient for MRI [34,35] and should be performed with due care by a specialist radiologist before qualifying a patient for MRI. The literature provides studies concerning the use of artificial neural networks to identify different types of foreign bodies on radiographs. Kim et al. proposed a model that detects cardiac implantable electronic devices (CIEDs) and characterizes their parameters. The main aim was to create a tool to help classify patients with such implants for MRI. This is especially important when there are no documentation or problems obtaining device information from the patient, such as when the patient is unconscious [36]. Advances in the refinement and miniaturization of cardiac rhythm management devices have led to the widespread use of safer and less fail-safe implantable leadless electrical devices (LLIEDs) [37]. For most devices of this type, MRI is referred to as “conditional”, i.e., the test is safe under certain conditions, as specified by the device manufacturer [38]. However, due to the small size and common factors that interfere with the evaluation of radiographs (such as suboptimal inspection techniques, motion artifacts, or other impermeable objects), the presence of the device can be ignored. The resulting model developed by Kim et al. took less than 7 min to analyze about 8000 images in the training phase, resulting in about 19 CXRs per second [31]. In comparison, the model created for our study analyzes as many as 250 X-ray images per second. A study by Thurston et al. aimed to create a similar tool, which achieved 99.67% accuracy on the test set [39]. Also, models developed by White et al. to support detection and identification of LLIED on CXRs performed prior to scheduled MRI scans achieved accuracies of 94.5% and 95%, respectively. Safety-level categorization was high during both testing (AUC ≥ 0.98 and ≥0.99, respectively) and trialing (accuracy 98% and 97%, respectively) [36]. Comparatively, our model achieved an accuracy of >99%.

Another type of foreign object for which artificial neural networks have been designed to recognise is shoulder joint endoprostheses. Joint degeneration, recognized by the WHO as a disease of civilization, has contributed to the widespread performance of endoprostheses, which, in Poland, reaches about 90,000 procedures per year, which is associated with the emergence of a large group of patients, with the implant also being an artifact on the X-ray image [40]. Sultan et al. proposed using AI to develop a personalized approach for patients just qualified for shoulder joint prosthesis revision. The standard method usually used in the absence of implant documentation, i.e., the comparison of radiographs of the patient with radiographs of the shoulder prosthesis by a specialist, is time consuming and error prone. The results of the IMFC-Net model they developed were promising—an average accuracy of 89.09%, a precision rate of 89.54%, a recall rate of 86.57%, and an F1 score of 87.94% [23]. However, our model demonstrated the ability to detect shoulder endoprostheses with an accuracy of 98.80%. Another study by Deshpande et al. showed AUC of 0.997 for pacemakers and 0.988 for jewelry (necklaces and earrings). Our results for pacemakers and necklaces were quite similar (0.995 and 0.988, respectively) [41].

The use of foreign body detection models does not end there. It is important to keep in mind the risk of radiologists misinterpreting additional elements present on radiographs and attributing to them a pathological etiology or, on the contrary, blurring the pathological image through the foreign body. In their work, Dick et al. described the case of a patient who had a rupture of one of her breast implants that resulted in migration of silicone, the image of which suggested a nodular lesion in the lung [15]. An entirely different situation has been described by Fitter and Cowie: visible artifacts on radiographs, a consequence of the presence of breast implants, obscuring pathological lesions present in the lung; this is only seen on x-rays after the implant is removed [42].

Research to date has primarily focused on AI’s use in identifying objects in X-rays within specific fields. Targeted tools have shown promising results in various sectors. However, the ultimate goal is to develop a comprehensive model capable of detecting and describing multiple foreign elements in a single patient during one examination. Our model takes a significant step towards this by simultaneously detecting four different elements. To improve our neural network’s usability, we should use a larger, diverse dataset during training. Including more low-quality radiographs for continuous learning and retraining will enhance the tool’s accuracy and reliability. Moreover, adding new foreign objects to the re-trained model opens up the possibility to determine the correctness of the insertion of certain implantable elements like endotracheal tubes or the exact type of endoprosthesis or ICD. Identifying foreign objects on X-rays requires experience, and it is also a time-consuming process. Manual labeling of such a large amount of data can involve the possibility of misidentifications. Most of these are corrected by the verification team. The additional screening can, therefore, reduce the likelihood of worthless data being fed into the network. Another solution is to use an auxiliary network model, verifying the input image. Despite the interest in AI, its direct implication in the medical field is a drawn-out process that is treated with a great deal of distrust. As the technical solutions that are put into clinical use must be of the highest quality, the following tool can serve as an aid to the work of the radiologist, who will still remain responsible for the final diagnosis.

The pursuit of this research article was challenged by several limitations, primarily stemming from the limited availability of CXR featuring foreign bodies. This scarcity precluded the creation of a comprehensive test dataset, although it is noteworthy that this constraint did not adversely impact the ultimate findings of our study. Furthermore, the absence of a sufficient pool of comparable studies in the field posed an additional constraint, restricting our ability to make direct comparisons with other researchers who have explored similar topics.

## 6. Conclusions

The impressive results obtained by our model suggest its usefulness in the detection of artifacts in X-ray images, both the type of artifact and its location. To further enhance the accuracy and reliability of the discussed tool, it is worth implementing a larger amount of more diverse image data in model training. Nevertheless, the present results suggest the possibility of imminent acceptance of the network in clinical use. Such validation would make it possible to use it as an advisory tool used to signal the occurrence of artifacts without engaging the attention of a medical specialist. The ability to detect objects in low-quality or abnormal images highlights the strength of our network. Automating the artifact detection-process using AI would guarantee the accuracy of object classification, reduce the risk of missing objects and significantly speed up the creation of X-ray descriptions. The time saved on the identification and description of the artifact could be spent on the analysis of other pathologies visible in the image. Further research is necessary to ensure the safe use of the model in clinical practice and its potential to enhance the work of radiology specialists.

## Data Availability

Dataset used: https://www.kaggle.com/datasets/nih-chest-xrays/data, Other data is available from the authors and is made available on request to interested parties.
